# Amino acid homeostasis in the kidney: Physiological roles and pathological dysregulation

**DOI:** 10.14814/phy2.71029

**Published:** 2026-08-03

**Authors:** Shuo Liu, Caifeng Shi, Yang Zhou, Chunsun Dai

**Affiliations:** ^1^ Center for Kidney Diseases The Second Affiliated Hospital of Nanjing Medical University Nanjing China

**Keywords:** amino acid metabolism, branched‐chain amino acids, chronic kidney disease, kidney physiology, metabolic reprogramming, uremic toxins

## Abstract

Amino acids are fundamental to life as protein building blocks and key regulators of metabolism and signaling. The kidney plays a critical, yet underappreciated, role in amino acid homeostasis through three interconnected pillars: selective glomerular filtration, efficient tubular reabsorption, and metabolic processing, which includes de novo synthesis and interconversion of amino acids, as well as their catabolism for energy production and gluconeogenesis. These processes are tightly coupled to systemic acid–base regulation, gluconeogenesis, and whole‐body nitrogen clearance. When kidney function declines, the resulting alterations in amino acid profiles are not merely passive markers of reduced filtration but are increasingly recognized as potential mediators that may actively contribute to disease progression, as supported by a growing body of preclinical and clinical evidence. This Review synthesizes current knowledge on renal amino acid homeostasis and proposes four dysregulation patterns in kidney disease: metabolic rewiring, branched‐chain amino acids paradox, uremic toxin accumulation, and organelle dysfunction. We further discuss emerging therapeutic strategies aimed at restoring amino acid homeostasis, including dietary modulation, pharmacological targeting of metabolic enzymes and transporters, and microbiome‐directed interventions. Finally, we identify key unanswered questions that should guide future research in this rapidly evolving field.

## 1 INTRODUCTION

Amino acids are indispensable to life, acting not only as the raw materials for protein synthesis but also as key regulators of metabolism, signaling, and gene expression. Maintaining amino acid homeostasis, defined as the dynamic equilibrium between synthesis, degradation, uptake, and excretion, is essential for organismal health. Disruption of this balance is increasingly recognized as a driver, rather than merely a consequence, of various disease states (Brosnan & Brosnan, 2006).

The liver and skeletal muscle have traditionally been viewed as the principal organs governing amino acid metabolism. However, accumulating evidence over the past three decades has revealed that the kidney also plays a crucial, and often neglected function within this regulatory framework (Garibotto et al., 2010). The kidney contributes to amino acid balance through three interconnected mechanisms: selective glomerular filtration, efficient tubular reabsorption, and active metabolic processing. This latter pillar encompasses the de novo synthesis of several nonessential amino acids (e.g., serine and arginine), their interconversion (e.g., the hydroxylation of phenylalanine to tyrosine), and their catabolism, which fuels renal gluconeogenesis and provides energy for tubular transport processes (Garibotto et al., 2010; Knol et al., 2024; van de Poll et al., 2004). This active metabolic processing also contributes to the hydrolysis of filtered peptides, thereby influencing the free amino acid pool available for systemic use. Through these processes, renal amino acid metabolism is tightly coupled to systemic acid–base regulation, gluconeogenesis, and whole‐body nitrogen clearance (Knol et al., 2024; Weiner et al., 2015).

Recent advances have substantially expanded our understanding of how specific amino acid metabolic pathways become dysregulated in kidney disease and actively contribute to its progression. Preclinical evidence has demonstrated that in autosomal dominant polycystic kidney disease (ADPKD), cystic epithelial cells exhibit a metabolic reprogramming characterized by increased dependence on glutamine and asparagine to fuel the tricarboxylic acid (TCA) cycle and pyrimidine synthesis (Clerici et al., 2024; Podrini et al., 2018). Similarly, studies in animal models have shown that in diabetic kidney disease (DKD), defective catabolism of branched‐chain amino acids (BCAAs) in podocytes promotes apoptosis and metabolic reprogramming (Zhao, Sun, et al., 2025). Of translational relevance, elevated urinary BCAA excretion predicts rapid chronic kidney disease (CKD) progression (Liu et al., 2026; Mutter et al., 2022). Notably, it is important to recognize that multiple factors, including hyperglycemia itself, also drive cellular senescence in this complex pathological context. Furthermore, the tryptophan‐derived uremic toxin indoxyl sulfate (IS) activates the aryl hydrocarbon receptor (AhR), promoting renal senescence and fibrosis by suppressing PGC1α‐mediated mitochondrial biogenesis (Mutter et al., 2022; Schroeder et al., 2010). Conversely, beneficial microbial metabolites of tryptophan, such as indole‐3‐propionic acid (IPA), are decreased in DKD and exert protective effects by enhancing SIRT1‐PGC1α signaling in glomerular endothelial cells (Roager & Licht, 2018; Zeng et al., 2025).

Epigenetic and posttranslational mechanisms have emerged as key links between amino acid metabolism and kidney injury. Methionine restriction can alleviate renal fibrosis by epigenetically repressing the TGF‐β‐Smad3‐Hoxc8/P‐TEFb axis (Liu et al., 2025). Lactate‐derived lysine lactylation, particularly of acyl‐CoA synthetase family member 2 (ACSF2), contributes to mitochondrial dysfunction in diabetic nephropathy (Chen et al., 2024; Zhang et al., 2019). Impaired serine biosynthesis via phosphoglycerate kinase 1 (PGK1) downregulation drives podocyte senescence (Hu et al., 2025), and specific amino acid transporters, such as SLC25A45, are required for mitochondrial uptake of trimethyllysine to drive carnitine biosynthesis (Dias et al., 2025). The interplay between amino acid metabolism and therapeutic interventions has also gained attention; infusion of amino acids reduced the occurrence of acute kidney injury (AKI) after cardiac surgery (Jufar et al., 2020; Landoni et al., 2024), and SGLT2 inhibition modulates amino acid metabolism through both renal and gut microbiome effects (Billing et al., 2024; Maekawa et al., 2025).

While comprehensive recent reviews have elegantly summarized the fundamental aspects of renal amino acid handling (Knol et al., 2024), the present review aims to provide a distinct, pathophysiology‐focused framework. Specifically, we move beyond a descriptive account of metabolism to propose a novel classification of the “patterns of dysregulation” (metabolic rewiring, BCAA paradox, uremic toxin accumulation, and organelle dysfunction) that are shared across different kidney diseases. Furthermore, we critically appraise the latest translational evidence, including the PROTECTION trial and emerging microbiome‐directed therapies, offering a forward‐looking perspective on therapeutic opportunities that are not the central focus of existing literature.

## 2 THE KIDNEYS AS CENTRAL REGULATORS OF AMINO ACID HOMEOSTASIS

The kidney's role in amino acid homeostasis extends far beyond simple filtration and excretion. Rather, the kidney functions as an active metabolic hub that integrates three interconnected processes, such as selective filtration, efficient reabsorption, and active metabolic processing (encompassing synthesis, interconversion, and catabolism) to maintain systemic amino acid balance (Brosnan & Brosnan, 2006; Knol et al., 2024). These three pillars work in concert to retain essential nutrients, eliminate waste products, and supply specific amino acids to peripheral tissues.

### 2.1 Selective filtration

The initial step in renal amino acid handling occurs at the glomerulus, where amino acids are freely filtered due to their low molecular weight (Knol et al., 2024). However, the extent of filtration is not uniform across all amino acid species. Hydrophobic amino acids, which have a higher propensity to bind plasma proteins, exhibit lower filtration fractions compared to their hydrophilic counterparts (Knol et al., 2024). This protein‐binding differential creates a natural selection mechanism that influences the relative delivery of individual amino acids to the proximal tubule. Approximately 50–70 grams of amino acids are filtered daily in the human kidney, representing a substantial metabolic load that must be efficiently managed to prevent nutrient loss (Garibotto et al., 2010; Knol et al., 2024).

### 2.2 Efficient reabsorption

The proximal tubule is the primary site for amino acid reabsorption, recovering approximately 97–99% of filtered amino acids through a sophisticated network of apical and basolateral transporters (Knol et al., 2024; Verrey et al., 2009). This process is highly efficient but also energy‐intensive, relying on sodium‐coupled transport mechanisms and mitochondrial oxidative phosphorylation (Bhargava & Schnellmann, 2017).

The SLC (solute carrier) family of transporters mediates the bulk of amino acid reabsorption. Key apical transporters include SLC6A19 (B^0^AT1) for neutral amino acids (Xu et al., 2024), SLC3A1/SLC7A9 for cystine and dibasic amino acids (Fotiadis et al., 2013), and SLC1A1 (EAAC1) for glutamate and aspartate (Canul‐Tec et al., 2017). Basolateral exit is mediated by transporters such as SLC7A8 (LAT2) and SLC3A2 (4F2hc), which facilitate the release of amino acids into the circulation (Fotiadis et al., 2013; Verrey et al., 2004). The overlapping substrate specificities of these transporters provide functional redundancy and resilience against single‐transporter defects (Broer & Broer, 2017).

The clinical importance of this reabsorptive machinery is underscored by inherited aminoacidurias. Mutations in SLC3A1 or SLC7A9 cause cystinuria, characterized by excessive urinary excretion of cystine and dibasic amino acids, leading to recurrent nephrolithiasis (Font‐Llitjos et al., 2005). Similarly, mutations in SLC6A19 result in Hartnup disorder, presenting with neutral aminoaciduria and pellagra‐like symptoms due to impaired tryptophan reabsorption (Seow et al., 2004). These genetic disorders highlight the nonredundant roles of specific transporters in maintaining amino acid balance.

Beyond hereditary disorders, acquired transporter dysfunction contributes to kidney disease progression. In CKD, reduced expression of organic anion transporters OAT1 and OAT3 impairs the secretion of uremic toxins and certain amino acid metabolites (Wu et al., 2017). SGLT2 inhibition downregulates multiple apical transporters, including amino acid transporters, thereby reducing the reabsorptive burden on proximal tubule cells and protecting against glucotoxicity and hyperfiltration‐induced injury (Maekawa et al., 2025; Vallon & Thomson, 2020).

### 2.3 Metabolic processing: Synthesis, interconversion, and catabolism

Perhaps the most underappreciated function of the kidney is its capacity for active metabolic processing of amino acids, which encompasses not only their de novo synthesis and interconversion but also their catabolism for energy production and gluconeogenesis (Knol et al., 2024). The kidney is a primary site for the production of serine, arginine, and tyrosine, contributing substantially to circulating levels of these amino acids, particularly during fasting or metabolic stress. Concurrently, the kidney oxidizes amino acids such as glutamine and leucine to fuel the TCA cycle and support tubular transport functions, highlighting its dual role as both a supplier and a consumer of amino acids.

Serine synthesis is a notable example of renal metabolic specialization. The kidney produces high amounts of serine from the glycolytic intermediate 3‐phosphoglycerate via the phosphorylated pathway, involving enzymes such as PHGDH and PSAT1 (Chen et al., 2022; Holecek, 2022). During fasting, when dietary serine is limited, the kidneys become the primary source of circulating serine (Lowry et al., 1987). Impaired serine biosynthesis has been implicated in podocyte senescence and DKD progression. Hu et al. demonstrated that Ang II downregulates PGK1 via FOXA1, leading to reduced serine biosynthesis, mitochondrial dysfunction, and podocyte senescence (Hu et al., 2025). Serine supplementation ameliorated these pathological changes, highlighting the therapeutic potential of targeting the PGK1‐serine axis (Handzlik et al., 2023).

Arginine synthesis represents another critical renal function. The kidney is the body's primary source of systemic arginine produced from citrulline, a process involving ASS1 and ASL (Dhanakoti et al., 1990; Trott et al., 2018). This pathway is essential for maintaining nitric oxide (NO) production, which regulates vascular tone and glomerular hemodynamics (Carlstrom, 2021). In CKD, reduced arginine bioavailability contributes to endothelial dysfunction and cardiovascular complications (Duranton et al., 2014).

Tyrosine synthesis from phenylalanine is also mediated by the kidney via phenylalanine 4‐hydroxylase (Tessari et al., 1999). Circulating tyrosine concentrations are lower in patients with CKD than in healthy controls, reflecting reduced renal conversion capacity (Boirie et al., 2004). Tyrosine deficiency may contribute to catecholamine dysregulation and metabolic disturbances in advanced kidney disease.

Beyond anabolism, the kidney actively participates in amino acid catabolism. For instance, the kidney is a major site for the oxidation of branched‐chain amino acids (BCAAs) and glutamine, which provides critical anaplerotic substrates for the TCA cycle and supports the high energy demands of proximal tubular transport (Harper et al., 1984; Stumvoll et al., 1999). Renal glutamine catabolism, in particular, is tightly coupled to acid–base homeostasis, as its deamidation by glutaminase generates ammonium for urinary excretion and bicarbonate for systemic pH buffering (Weiner et al., 2015). This catabolic arm of metabolism is as integral to renal function as synthesis and reabsorption, yet it is often overlooked in discussions of amino acid homeostasis.

### 2.4 Connecting amino acid metabolism to systemic homeostasis

The three pillars of renal amino acid handling are tightly coupled to broader systemic homeostatic processes. First, acid–base balance is critically dependent on renal glutamine metabolism. In response to acidosis, proximal tubule cells upregulate GLS1, which deamidates glutamine to glutamate and ammonium, generating bicarbonate that is returned to the circulation (Stumvoll et al., 1999). This process accounts for a substantial fraction of the body's ability to maintain pH homeostasis (van de Poll et al., 2004).

Second, energy metabolism is intimately linked to amino acid handling. Amino acids serve as anaplerotic substrates for the TCA cycle, particularly in the proximal tubule, where glutamine‐derived α‐ketoglutarate fuels oxidative phosphorylation (Stumvoll et al., 1999; Yang et al., 2014). Renal gluconeogenesis, which contributes 20–25% of systemic glucose production during fasting, relies on amino acid substrates such as glutamine and alanine (Stumvoll et al., 1999).

Third, nitrogen balance is coordinated between the kidney and liver. While the liver disposes of nitrogen through the urea cycle, the kidney handles approximately 5% of nitrogen excretion via a glutamine‐dependent pathway, with the remainder excreted as urea (Knol et al., 2024).

Emerging evidence also highlights the role of mitochondrial amino acid transport in maintaining renal homeostasis. Dias et al. identified SLC25A45 as a mitochondrial transporter required for the uptake of methylated amino acids, particularly trimethyllysine, to drive carnitine biosynthesis (Dias et al., 2025). A missense variant (R285C) in SLC25A45 stabilizes the transporter and associates with altered methylated amino acid levels in human plasma, suggesting that mitochondrial amino acid handling is a previously unappreciated determinant of renal metabolic health.

Finally, the gut–kidney axis has emerged as an important modulator of amino acid homeostasis. The gut microbiota metabolizes tryptophan into indole and indole derivatives, which are absorbed and further metabolized in the liver to uremic toxins such as IS (Tan et al., 2017). These toxins accumulate in CKD and activate AhR, promoting renal senescence and fibrosis (Xie et al., 2024). Conversely, certain gut bacteria, such as Bifidobacterium species, can salvage indole to synthesize indole‐3‐lactic acid (ILA), an immunomodulatory metabolite with potential beneficial activities (Yong et al., 2024). Dietary fiber intake directs microbial tryptophan metabolism by altering bacterial competition for tryptophan (Sinha et al., 2024).

**FIGURE 1:**
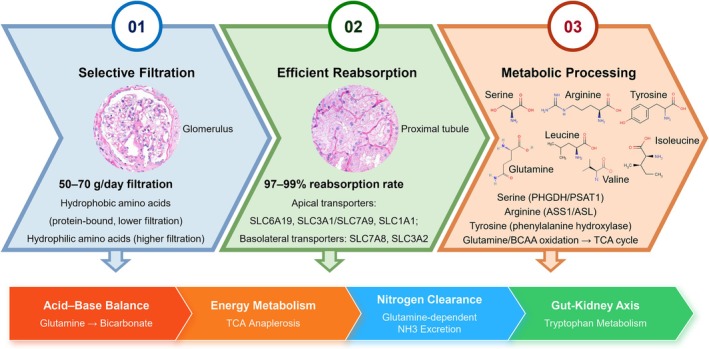
The three pillars of renal amino acid homeostasis. The kidney maintains systemic amino acid balance through three integrated mechanisms. Pillar I (Selective filtration): Approximately 50–70 g of amino acids are filtered daily at the glomerulus. Hydrophobic amino acids, which bind plasma proteins, exhibit lower filtration fractions than hydrophilic ones. Pillar II (Efficient reabsorption): The proximal tubule reabsorbs 97–99% of filtered amino acids via apical (SLC6A19, SLC3A1/SLC7A9, SLC1A1) and basolateral (SLC7A8, SLC3A2) transporters. Pillar III (Metabolic processing: Synthesis, interconversion, and catabolism): The kidney synthesizes serine (via PHGDH/PSAT1), arginine (via ASS1/ASL), and tyrosine (via phenylalanine hydroxylase); it also oxidizes glutamine and branched‐chain amino acids (BCAAs) to provide anaplerotic substrates for the TCA cycle, supporting tubular energy demands. These three pillars are coupled to systemic functions including acid–base balance (glutamine is converted by GLS1 to generate bicarbonate), energy metabolism (anaplerotic TCA cycle substrates derived from amino acid catabolism), nitrogen clearance (glutamine‐dependent ammonia excretion), and the gut–kidney axis (tryptophan metabolism). Figure 1 was created by the authors using Microsoft PowerPoint. No third‐party copyrighted materials were used.

In summary, the kidney maintains amino acid homeostasis through three integrated pillars: selective filtration, efficient reabsorption, and metabolic processing, tightly coupled to systemic acid–base balance, energy metabolism, and nitrogen clearance. These processes are illustrated in Figure 1. Disruption of any pillar contributes to the pathogenesis of kidney disease, as will be discussed in the following sections.

## 3 PATTERNS OF AMINO ACID DYSREGULATION IN KIDNEY DISEASE

**FIGURE 2:**
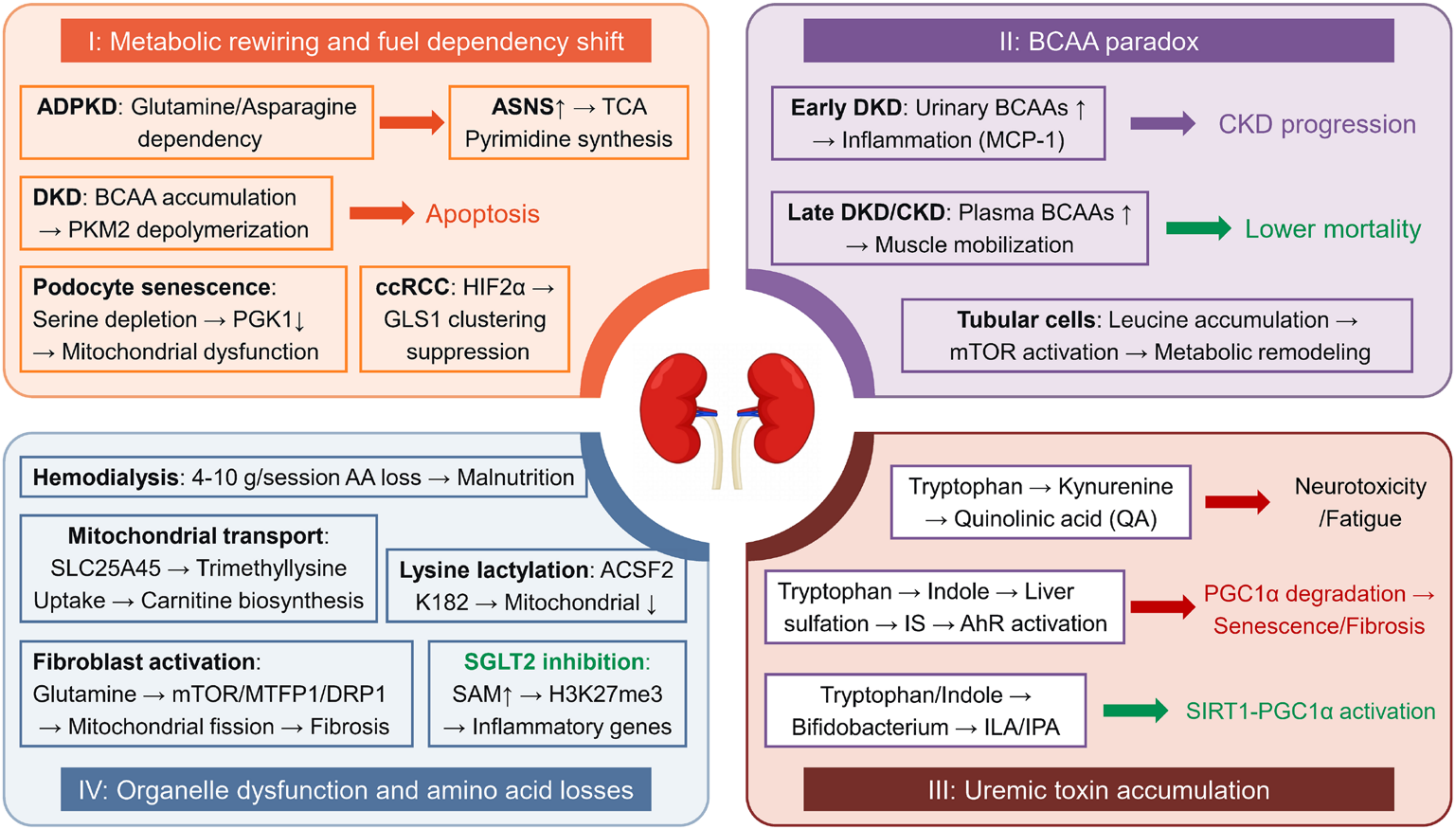
Four patterns of amino acid dysregulation in kidney disease. When kidney function declines, amino acid homeostasis fails through four interconnected patterns. Pattern I (Metabolic rewiring and fuel dependency shift): In ADPKD, cystic epithelial cells become dependent on glutamine and asparagine via ASNS upregulation. In DKD, BCAA accumulation drives PKM2 depolymerization and podocyte apoptosis. Serine depletion contributes to podocyte senescence. In ccRCC, HIF2α prevents GLS1 clustering, maintaining low glutaminase activity. Pattern II (BCAA paradox): Elevated urinary BCAAs predict CKD progression in early DKD, whereas higher plasma BCAAs associate with lower mortality in advanced disease. Leucine accumulation also activates mTOR signaling in tubular cells. Pattern III (Uremic toxin accumulation): Tryptophan‐derived metabolites, including indoxyl sulfate (IS) and quinolinic acid (QA), accumulate in CKD, promoting renal senescence, fibrosis, and neurotoxicity via the AhR‐PGC1α and kynurenine pathways. Beneficial metabolites (indole‐3‐lactic acid, ILA; indole‐3‐propionic acid, IPA) are reduced. Pattern IV (Organelle dysfunction and amino acid losses): 4 to 10 grams of amino acids per session, depending on dialyzer type and nutritional status. Mitochondrial dysfunction occurs via SLC25A45‐dependent carnitine biosynthesis defects, ACSF2 lysine lactylation, and dysregulated glutamine metabolism in fibroblasts. Methionine metabolism represents a double‐edged sword: While SGLT2 inhibitors exert protective effects through SAM‐dependent epigenetic regulation (H3K27me3), excessive methionine‐derived sulfate contributes to metabolic acidosis and impaired renal S‐adenosylhomocysteine (SAH) clearance promotes cardiovascular toxicity in CKD. Figure 2 was created by the authors using Microsoft PowerPoint. No third‐party copyrighted materials were used.

Under physiological conditions, the kidney maintains amino acid homeostasis through the integrated three‐pillar system described above. However, when kidney function declines, whether in acute injury or chronic disease, these finely tuned processes become dysregulated. The resulting alterations in amino acid profiles are not passive markers of reduced filtration but actively contribute to disease progression (Knol et al., 2024). Based on a synthesis of recent evidence, we propose that amino acid dysregulation in kidney disease manifests through four distinct but interconnected patterns as outlined in Figure 2.

### 3.1 Metabolic rewiring and fuel dependency shift

A hallmark of cellular transformation in kidney disease is the reprogramming of core metabolic pathways, wherein cells shift from flexible fuel utilization to rigid dependency on specific amino acids. This pattern is most prominently observed in ADPKD and DKD.

#### 3.1.1 ADPKD: Glutamine and asparagine dependency

In preclinical models of ADPKD, cystic epithelial cells exhibit a metabolic reprogramming reminiscent of the Warburg effect in cancer (Rowe et al., 2013). Unlike healthy tubular epithelial cells, which use pyruvate to fuel the TCA cycle, PKD cells rely on glutamine to support growth, maintain mitochondrial membrane potential, and fuel the TCA cycle (Flowers et al., 2018; Podrini et al., 2018). This glutamine dependency is driven by asparagine synthetase (ASNS), which deamidates glutamine to glutamate while synthesizing asparagine from aspartate (Lomelino et al., 2017). Silencing Asns in vitro impairs glutamine utilization, compromises cell survival, and reduces proliferation (Clerici et al., 2024). Mechanistically, ASNS upregulation in ADPKD is mediated by the amino acid response branch of the integrated stress response, specifically via GCN2‐dependent activation of ATF4. Targeting ASNS with antisense oligonucleotides in a slowly progressive PKD mouse model led to a drastic reduction in total kidney volume and a prominent rescue of renal function. Metabolomic profiling revealed that ASNS silencing rescued multiple metabolic pathways, particularly de novo pyrimidine synthesis (Clerici et al., 2024).

#### 3.1.2 DKD: BCAA‐driven podocyte metabolic reprogramming

In DKD, BCAAs drive podocyte injury through a distinct mechanism involving pyruvate kinase M2 (PKM2). In patients with DKD and in db/db mice, Zhao and colleagues identified defective BCAA catabolism within podocytes, as evidenced by heightened phosphorylation and consequent inactivation of the branched‐chain ketoacid dehydrogenase (BCKD) complex. This metabolic impairment results in the buildup of BCAAs, which in turn triggers the disassembly and functional suppression of PKM2. The depolymerized form of PKM2 not only inhibits glucose‐driven oxidative phosphorylation but also shifts glucose utilization toward serine biosynthesis and folate metabolism. Moreover, depolymerized PKM2 translocates to the nucleus in association with DDIT3, where it functions as a co‐transcriptional partner to augment DDIT3‐mediated gene activation. This leads to increased expression of CHAC1 and TRIB3, thereby directly inducing apoptotic cell death in podocytes. Notably, pharmacological interventions, either PKM2 activation via TEPP46 or restoration of BCAA breakdown using the BDK inhibitor BT2, effectively attenuated podocyte damage and improved renal pathology in diabetic mouse models (Zhao, Sun, et al., 2025).

#### 3.1.3 Serine depletion and podocyte senescence

Another manifestation of metabolic rewiring is the depletion of serine in podocytes under stress conditions. According to the study by Hu and colleagues, angiotensin II (Ang II) suppresses PGK1 expression via the transcription factor FOXA1. This downregulation results in diminished serine production, impaired mitochondrial function, and accelerated podocyte senescence. Knocking down PGK1 reproduced these cellular abnormalities. Conversely, replenishing serine or overexpressing PGK1 mitigated Ang II‐induced mitochondrial damage, disruption of the actin cytoskeleton, and senescence‐related features both in cultured podocytes and in mouse models of chronic kidney disease. Further mechanistic investigation revealed that PGK1 binds to and stabilizes KRT1, thereby preserving cytoskeletal integrity through a mechanism independent of its catalytic activity (Hu et al., 2025). This study establishes the PGK1–serine axis as a critical determinant of podocyte homeostasis.

#### 3.1.4 HIF2α‐mediated suppression of glutaminase clustering in ccRCC

Clear cell renal cell carcinoma (ccRCC) shares certain metabolic characteristics with ADPKD. In this cancer type, HIF2α suppresses the assembly of glutaminase (GLS1) into clusters within mitochondria, thus preserving low GLS enzymatic activity and shielding ccRCC cells from cell death induced by glutamine withdrawal. In most other cell types, deprivation of glutamine promotes GLS1 clustering, which substantially boosts its enzymatic function and triggers apoptosis. By contrast, in ccRCC cells, HIF2α blocks this clustering event independently of its role in transcriptional regulation. When a constitutively clustering GLS1 mutant was introduced to enforce clustering, high GLS activity was restored, apoptosis was enhanced, and tumor growth was suppressed in vivo (Zhao, Demczyszyn, et al., 2025).

#### 3.1.5 AKI and amino acid metabolism

In AKI, metabolic rewiring also involves amino acid pathways. In a multinational, double‐blind randomized trial involving 3511 patients undergoing cardiac surgery, Landoni and colleagues reported that intravenous administration of a balanced amino acid solution lowered the incidence of AKI from 31.7% in the placebo arm to 26.9% in the amino acid group (relative risk: 0.85; 95% confidence interval: 0.77–0.94; *p* = 0.002). The proportion of patients developing Stage 3 AKI was 1.6% in the amino acid group, compared with 3.0% in the placebo group (Landoni et al., 2024). This trial provides strong clinical evidence that amino acid administration can protect against AKI, likely through the recruitment of renal functional reserve.

### 3.2 The paradox of branched‐chain amino acids

Among all amino acids, BCAAs exhibit the most complex and context‐dependent relationship with kidney disease. Across different disease stages and patient populations, BCAAs demonstrate seemingly contradictory effects, predicting disease onset in some contexts while associating with favorable outcomes in others (Lotta et al., 2016; Palmer et al., 2015; Wang et al., 2011; Welsh et al., 2018).

#### 3.2.1 BCAAs as predictors of DKD onset

A large prospective study enrolling 1868 patients with type 2 diabetes revealed that elevated urinary levels of valine, leucine, and isoleucine were each independently linked to a heightened risk of progressive CKD. Following adjustment for conventional clinical risk factors, each one‐standard‐deviation increase in urinary valine, leucine, or isoleucine corresponded to a 1.29‐fold, 1.31‐fold, and 1.29‐fold higher risk of the composite kidney endpoint, respectively. Mediation analysis further indicated that urinary MCP‐1 accounted for 47–58% of the effect of BCAAs on renal outcomes, implying that disrupted BCAA handling within the kidney fuels inflammatory responses and thereby accelerates CKD progression (Liu et al., 2026). These findings were externally validated in the CRIC study (Kwan et al., 2020).

#### 3.2.2 BCAAs as markers of favorable outcomes in advanced disease

Counterintuitively, among individuals with established DKD or type 1 diabetes, elevated BCAA concentrations have been linked to reduced mortality and a decelerated rate of kidney function decline. Similarly, data from the ADVANCE trial, which enrolled participants with type 2 diabetes, showed that higher circulating levels of leucine and valine correlated with a diminished risk of death (Welsh et al., 2018). Likewise, in a separate cohort comprising 637 individuals with type 1 diabetes, elevated BCAA levels correlated with a reduced risk of the composite renal outcome, which included progression to kidney failure, decline in eGFR, and death from any cause (Tofte et al., 2019). Additional investigations have found that elevated plasma BCAA concentrations are inversely correlated with both the development and the presence of CKD in individuals with type 2 diabetes (de Klerk et al., 2024).

This paradox may be explained by several nonmutually exclusive mechanisms. First, elevated BCAAs in early disease may reflect insulin resistance and metabolic dysfunction, whereas in advanced disease, BCAAs may be mobilized from muscle wasting and serve as alternative energy substrates (Nagata et al., 2020). Second, BCAA catabolism is tissue‐specific; impaired catabolism in the kidney may lead to local BCAA accumulation and toxicity, while systemic BCAA elevation may reflect preserved hepatic and muscle function (Choi et al., 2024). Third, BCAAs may exert direct protective effects in certain contexts, such as reducing oxidative stress or preserving mitochondrial function (Mi et al., 2015).

#### 3.2.3 Leucine‐mediated mTOR activation in tubular cells

Beyond DKD, leucine accumulation activates the mTOR signaling pathway in renal tubular cells. Shi et al. demonstrated that defective leucine degradation in diabetic kidneys, mediated by upregulation of BCKDK, leads to leucine accumulation, which stimulates metabolic remodeling via the mTOR signaling pathway. Enhancing leucine degradation by genetic or pharmacological targeting of BCKDK relieved glucose‐induced metabolic remodeling and mitigated DKD in mouse models. In diabetic mice, dietary leucine restriction led to marked reductions in albuminuria, renal hypertrophy, and lipid deposition. Furthermore, among diabetic patients, a faster deterioration of kidney function was associated with an elevated urinary leucine‐to‐creatinine ratio, pointing to leucine as a possible causal contributor and a promising therapeutic target for DKD (Shi et al., 2025).

### 3.3 Accumulation of uremic toxins

The progressive decline in kidney function leads to the accumulation of amino acid‐derived metabolites that are normally excreted in urine. Among these, tryptophan‐derived uremic toxins have received the most attention due to their direct pathogenic effects on the kidney and cardiovascular system (Vanholder et al., 2014).

#### 3.3.1 Indoxyl sulfate and the AhR–PGC1α Axis

IS, a tryptophan metabolite generated by gut microbial tryptophanase and subsequently sulfated in the liver, is a prototypical uremic toxin (Koppe et al., 2018). Xie and colleagues reported that IS upregulates and activates AhR in tubular epithelial cells, thereby driving renal senescence and fibrosis. Conditional knockout of AhR in renal tubules reduced these pathologies and also restored PGC1α‐mediated mitochondrial biogenesis in IS‐treated CKD mouse kidneys. Mechanistically, AhR binds to PGC1α and accelerates its ubiquitin‐proteasomal degradation through its E3 ubiquitin ligase activity. Moreover, PGC1α overexpression attenuated IS‐induced cellular senescence and extracellular matrix deposition in cultured tubular epithelial cells (Xie et al., 2024). This study establishes the IS–AhR–PGC1α axis as a key driver of uremic toxicity.

#### 3.3.2 The kynurenine pathway and kidney–brain crosstalk

The kynurenine pathway, which accounts for over 95% of tryptophan degradation, generates multiple neuroactive metabolites that accumulate in CKD (Hsu & Tain, 2020). In a mouse model of rapid kidney failure, Saliba and colleagues applied spatial and bulk metabolomics and discovered that the most significantly disrupted pathway was tryptophan metabolism. The neurotoxic metabolite quinolinic acid was found to accumulate in brain ependymal cells, where its presence was linked to neuroinflammation and cellular death. In patients with stage 3b–5 chronic kidney disease, quinolinic acid levels were increased, and higher concentrations were associated with greater fatigue and poorer life quality (Saliba et al., 2025). These observations establish the kynurenine pathway as a connecting link among progressive kidney dysfunction, systemic inflammatory responses, and adverse neurological outcomes.

#### 3.3.3 The gut–kidney Axis and tryptophan metabolism

The gut microbiota plays a dual role in tryptophan metabolism. On one hand, microbial tryptophanase produces indole, which is converted to IS in the liver (Tan et al., 2017). On the other hand, certain gut bacteria, particularly Bifidobacterium species, can salvage exogenous indole to synthesize ILA, an immunomodulatory metabolite with potential beneficial activities (Yong et al., 2024). This microbial metabolic route, not described before, employs the β subunit of tryptophan synthase together with aromatic lactate dehydrogenase. Dietary fiber intake directs microbial tryptophan metabolism by altering bacterial competition for tryptophan. The fiber‐degrader Bacteroides thetaiotaomicron cross‐feeds monosaccharides to indole‐producing E. coli, leading to catabolite repression of the tryptophanase gene tnaA and reduced indole production. This makes more tryptophan available to Clostridium sporogenes, increasing production of ILA and IPA (Sinha et al., 2024).

#### 3.3.4 The gut–kidney Axis in SGLT2 inhibition

SGLT2 inhibitors modulate amino acid metabolism through both renal and gut microbiome effects. Through an integrated multiomics approach, Billing and colleagues demonstrated that SGLT2 inhibition suppresses the production of gut microbiota‐derived uremic toxins, including p‐cresol sulfate, consequently reducing both systemic exposure to these compounds and the burden on renal detoxification pathways (Billing et al., 2024). Maekawa and colleagues further showed that SGLT2 blockade preserves renal function through an S‐adenosylmethionine (SAM)‐dependent epigenetic silencing of pro‐inflammatory genes, uncovering a previously unrecognized axis that connects methionine metabolism, histone methylation, and kidney protection (Maekawa et al., 2025).

### 3.4 Organelle dysfunction and amino acid losses

Kidney disease, particularly in its advanced stages, leads to net loss of amino acids through multiple mechanisms, contributing to malnutrition, muscle wasting, and organelle dysfunction (Ikizler et al., 2002; Post et al., 2022).

#### 3.4.1 Amino acid losses in dialysis

Individuals undergoing hemodialysis experience a daily loss of amino acids, with recent studies reporting losses that can range from 4 to over 10 grams per session, depending on dialyzer type and nutritional status (Ikizler et al., 2002; Mafrici et al., 2026; Post et al., 2022). For instance, an analysis comparing 59 hemodialysis patients with 33 healthy kidney donors revealed that amino acid losses over 24 h amounted to 4 g in the dialysis group, compared with only 0.6 g in the control subjects (Post et al., 2022). Newer high‐flux dialyzers may further increase these losses, exacerbating the risk of protein‐energy wasting (Ikizler et al., 2002). Elevated plasma taurine levels correlated with a reduced likelihood of severe fatigue, implying that taurine administration could alleviate fatigue symptoms in individuals receiving hemodialysis (Post et al., 2022). However, the mechanisms underlying taurine dysregulation in CKD are complex and not fully understood. Unlike other amino acids, the kidney's capacity to reabsorb taurine is relatively low, and its plasma levels are largely determined by dietary intake and muscle release (Chesney, 1985). Therefore, the observed association with fatigue may reflect overall nutritional status rather than a specific renal handling defect.

#### 3.4.2 Mitochondrial amino acid transport

Beyond bulk amino acid loss, specific mitochondrial amino acid transporters are essential for maintaining organelle function. Dias et al. identified SLC25A45 as a mitochondrial transporter required for the uptake of methylated amino acids, particularly trimethyllysine, to drive carnitine biosynthesis. A missense variant (R285C) in SLC25A45 stabilizes the transporter and associates with altered methylated amino acid levels in human plasma, linking mitochondrial amino acid handling to systemic metabolic health (Dias et al., 2025).

#### 3.4.3 Lysine Lactylation and mitochondrial dysfunction

Chen et al. discovered that lysine lactylation, a lactate‐derived posttranslational modification, contributes to mitochondrial dysfunction in diabetic nephropathy. Integrative lactylome analysis of diabetic mouse kidneys revealed that the majority of lactylated proteins were localized to mitochondria. Among these, lactylation of the K182 site of ACSF2 promoted mitochondrial dysfunction in renal tubular epithelial cells. ACSF2 expression was notably increased in the kidneys of db/db mice and patients with diabetic nephropathy (Chen et al., 2024). This study identifies lysine lactylation as a previously unrecognized mediator of diabetic kidney injury.

#### 3.4.4 Restoring BCAA catabolism to improve kidney function

In preclinical models, Bollinger and colleagues showed that defective BCAA catabolism correlates with cardiorenal metabolic syndrome. Administration of the BDK inhibitor BT2 ameliorated urinary protein levels, renal enlargement, and kidney histological abnormalities in two distinct animal models. Moreover, combined treatment with BT2 and the SGLT2 inhibitor empagliflozin produced synergistic benefits on renal metrics as well as mitochondrial abundance and bioenergetic capacity (Bollinger et al., 2025).

#### 3.4.5 Glutamine metabolism and mitochondrial energy generation

Beyond BCAAs, glutamine metabolism plays a crucial role in renal fibroblast activation and fibrosis. In the unilateral ureteral obstruction model, Cai and colleagues found that withdrawing glutamine suppressed fibroblast activation and alleviated renal fibrosis. Glutamine turnover proved critical for preserving mitochondrial integrity and bioenergetics, as supplementation with α‐ketoglutarate partially counteracted the consequences of glutamine depletion. At the molecular level, glutamine metabolism influences mitochondrial fission during fibroblast activation by modulating the mTOR/MTFP1/DRP1 signaling axis (Cai et al., 2024).

#### 3.4.6 Methionine and SAM in kidney protection

Maekawa et al. demonstrated that SGLT2 loss or inhibition increases renal SAM levels, leading to epigenetic repression of inflammatory genes via H3K27 trimethylation. Metabolomic analysis revealed that methionine metabolism was altered in the kidneys of mice lacking SGLT2 function, and MAT2A inhibition abrogated kidney protection. SAM supplementation prevented the upregulation of NF‐κB‐related genes induced by high glucose in proximal tubular cells (Maekawa et al., 2025).

Conversely, methionine metabolism is a double‐edged sword in CKD. Methionine is rich in sulfur, and its oxidation generates sulfate, contributing to metabolic acidosis, a common complication in advanced kidney disease. Furthermore, the kidney plays a critical role in the clearance of S‐adenosylhomocysteine (SAH), a toxic byproduct of methylation reactions. In CKD, impaired renal SAH removal can lead to its accumulation, which is associated with increased cardiovascular risk and endothelial dysfunction (Garibotto et al., 2009). Thus, while methionine restriction may offer therapeutic benefits via epigenetic modulation, systemic accumulation of sulfur metabolites and SAH in CKD poses significant toxicity risks that warrant careful consideration.

The key amino acid metabolic pathways and their associated therapeutic targets across different kidney diseases are summarized in Table 1.

**TABLE 1:** Key Amino Acid Metabolic Pathways and Therapeutic Targets in Kidney Diseases.

Disease	Amino acid/Pathway	Key molecules	Dysregulation pattern	Therapeutic strategy	References
ADPKD	Glutamine, Asparagine	ASNS, ATF4, GCN2	Metabolic rewiring (fuel dependency)	ASNS antisense oligonucleotides	Clerici et al. (2024)
ADPKD	Leucine, Methionine	mTORC1	Metabolic rewiring	Dietary restriction	Liu et al. (2025)
DKD	BCAAs	PKM2, BCKDK, DDIT3	BCAA paradox (early vs. late disease)	BDK inhibitor (BT2), PKM2 activator (TEPP46)	Zhao, Sun, et al. (2025)
DKD	Serine	PGK1, FOXA1, KRT1	Serine depletion, podocyte senescence	Serine supplementation	Hu et al. (2025)
DKD / CKD	Tryptophan, Indoxyl sulfate	AhR, PGC1α	Uremic toxin accumulation	AhR antagonists, dietary fiber, probiotics	Xie et al. (2024)
DKD	Tryptophan, IPA	SIRT1, PGC1α	Loss of protective metabolite	IPA postbiotic supplementation	Zeng et al. (2025)
CKD	Methionine	Hoxc8, TGF‐β‐Smad3, P‐TEFb	Epigenetic dysregulation (H3K4me3/H3K36me3); sulfur load & SAH accumulation (toxic)	Methionine restriction	Garibotto et al. (2009); Liu et al. (2025)
CKD / AKI	Glutamine, BCAAs	GLS1, BCKDH	Metabolic rewiring (defective catabolism / impaired anaplerosis)	Targeting GLS1 or BCKDH; mitochondrial support	Bollinger et al. (2025); Cai et al. (2024); Stumvoll et al. (1999)
Diabetic nephropathy	Lysine lactylation	ACSF2	Mitochondrial dysfunction	Targeting ACSF2	Chen et al. (2024)
CKD / Hemodialysis	Multiple amino acids	SLC25A45	Amino acid losses	Taurine or BCAA supplementation	Dias et al. (2025)
ccRCC	Glutamine	GLS1, HIF2α	Metabolic rewiring (GLS1 clustering suppression)	GLS1 clustering promotion	Zhao, Demczyszyn, et al. (2025)

*Note*: Therapeutic strategies marked with “ASOs” refer to antisense oligonucleotides.

Abbreviations: ADPKD, autosomal dominant polycystic kidney disease; AhR, aryl hydrocarbon receptor; ASNS, asparagine synthetase; BCAAs, branched‐chain amino acids; BCKDK, branched‐chain ketoacid dehydrogenase kinase; ccRCC, clear cell renal cell carcinoma; CKD, chronic kidney disease; DKD, diabetic kidney disease; GLS1, glutaminase 1; HIF2α, hypoxia‐inducible factor 2‐alpha; IPA, indole‐3‐propionic acid; PGK1, phosphoglycerate kinase 1; PGC1α, peroxisome proliferator‐activated receptor gamma coactivator 1‐alpha; PKM2, pyruvate kinase M2.

## 4 THERAPEUTIC STRATEGIES AND UNRESOLVED QUESTIONS

The recognition that amino acid dysregulation actively drives kidney disease progression has opened new avenues for therapeutic intervention. Emerging strategies can be broadly classified into dietary modulation, pharmacological targeting of metabolic enzymes and transporters, and gut microbiome‐directed interventions.

### 4.1 Dietary modulation

Dietary interventions remain the most direct approach to modulate amino acid homeostasis. Low‐protein diets have long been recommended for patients with CKD to reduce glomerular hyperfiltration and uremic toxin generation. However, adherence is challenging, and concerns about protein‐energy wasting have prompted interest in more targeted amino acid restrictions.

Methionine restriction (MetR) has emerged as a particularly promising strategy. In a systematic screen of 15 amino acid‐restricted diets in a unilateral ureteral obstruction mouse model, MetR was the most effective intervention for reducing renal fibrosis. Mechanistically, MetR reduced H3K4me3 and H3K36me3 histone modifications at the Hoxc8 locus, thereby suppressing the TGF‐β‐Smad3‐Hoxc8/P‐TEFb profibrotic axis. Fibroblast‐specific Hoxc8 knockout protected mice from renal fibrosis, and elevated HOXC8 expression was observed in patients with IgA nephropathy and ADPKD (Liu et al., 2025).

BCAA restriction has shown protective effects in DKD models. Shi et al. demonstrated that restricting dietary leucine significantly decreased albuminuria, kidney hypertrophy, and lipid accumulation in diabetic mice (Shi et al., 2025). However, the optimal degree and timing of BCAA restriction require further investigation, given the paradoxical associations of BCAAs with outcomes in advanced disease (Mikolajetz et al., 2026).

Serine supplementation represents a different dietary strategy, replenishing a protective amino acid that becomes deficient in disease. Hu et al. found that serine supplementation restored mitochondrial function, reduced podocyte senescence, and attenuated glomerular injury in CKD mouse models (Hu et al., 2025). This aligns with human studies showing that circulating serine levels are reduced in CKD patients and correlate with cognitive decline and reduced eGFR (Iwata et al., 2023; Nakade et al., 2026). The PROTECTION trial demonstrated that intravenous infusion of a balanced amino acid mixture reduced AKI after cardiac surgery (Landoni et al., 2024), suggesting that amino acid supplementation is actively protective in specific clinical contexts.

Another dietary approach under investigation involves restricting aromatic amino acids. Barba and colleagues showed that a diet low in tyrosine, phenylalanine, and tryptophan decreased renal fibrosis, inflammatory markers, and urinary protein excretion to a degree comparable with a low‐protein diet, while achieving a more pronounced lowering of the uremic toxins p‐cresol sulfate and IS (Barba et al., 2021).

### 4.2 Pharmacological targeting of metabolic enzymes and transporters

BDK inhibitors enhance BCAA catabolism by inhibiting BCKDK. BT2 improved renal function and ameliorated kidney pathology in two cardiorenal metabolic syndrome animal models. Coadministration of BT2 with empagliflozin demonstrated additive effects on kidney parameters and mitochondrial function (Bollinger et al., 2025).

ASNS inhibitors target glutamine dependency in ADPKD. Antisense oligonucleotides against Asns in a PKD mouse model drastically reduced total kidney volume and rescued renal function. Combining ASNS inhibition with the glycolytic inhibitor 2‐deoxy‐D‐glucose further improved efficacy (Clerici et al., 2024).

In DKD models, PKM2 activators including TEPP46 have demonstrated kidney‐protective actions. Treatment with TEPP46 counteracted podocyte damage induced by BCAA supplementation, elevated pyruvate kinase activity, and normalized mitochondrial performance. The genetic ablation of PKM2 specifically in podocytes abolished the protective effect of TEPP46, confirming that its action is mediated through this target (Zhao, Sun, et al., 2025).

AhR antagonists may counteract the deleterious effects of IS. Renal tubule‐specific AhR knockout attenuated renal senescence and fibrosis (Xie et al., 2024). However, AhR also has physiological functions in immune regulation (Coumoul et al., 2026), so selective targeting of pathogenic AhR activation remains challenging.

Targeting mitochondrial dysfunction has emerged as a promising therapeutic frontier. Several bioactive compounds, such as resveratrol, coenzyme Q10, and certain polyphenols, have been shown to preserve mitochondrial integrity and function in preclinical CKD models, partly by modulating amino acid‐sensitive pathways like SIRT1‐PGC1α signaling (Cecchini et al., 2026). These interventions aim to restore bioenergetics and reduce oxidative stress, addressing a root cause of organelle dysfunction linked to amino acid imbalance.

SGLT2 inhibitors have revolutionized CKD treatment and also modulate amino acid metabolism. They reduce gut microbiome formation of uremic toxins (Jiang et al., 2024) and protect kidney function via SAM‐dependent epigenetic repression of inflammatory genes (Maekawa et al., 2025).

### 4.3 Microbiome‐directed interventions

Probiotic interventions have shown promise. Oral administration of Bifidobacterium longum reduced serum IS concentration in dialysis patients. Yong et al. discovered that Bifidobacterium species can salvage exogenous indole to synthesize ILA via a previously unknown microbial metabolic pathway (Yong et al., 2024).

Adding fiber to the diet shifts how gut microbes process tryptophan by changing how bacteria compete for this amino acid (Sinha et al., 2024). According to a meta‐analysis, increasing dietary fiber intake markedly lowers circulating IS levels in individuals receiving hemodialysis (Wathanavasin et al., 2025).

Postbiotic administration of beneficial tryptophan metabolites is another emerging approach. Zeng et al. demonstrated that IPA supplementation ameliorated albuminuria and mitigated mitochondrial impairments in DKD mice via the SIRT1‐PGC1α pathway. In patients with DKD, serum IPA levels were significantly reduced and correlated with clinical parameters (Zeng et al., 2025).

A comprehensive summary of emerging therapeutic strategies targeting amino acid metabolism, including dietary interventions, pharmacological agents, and microbiome‐directed approaches, is provided in Table 2.

**TABLE 2:** Therapeutic Strategies Targeting Amino Acid Metabolism in Kidney Disease.

Intervention type	Specific strategy	Mechanism of action	Disease/Model	Level of evidence	References
Dietary	Methionine restriction	Epigenetic repression of H3K4me3/H3K36me3 at Hoxc8 locus	UUO mouse (fibrosis model)	Preclinical	Liu et al. (2025)
Dietary	Leucine restriction	Inhibition of mTOR signaling	DKD mouse	Preclinical	Shi et al. (2025)
Dietary	Serine supplementation	Restoration of mitochondrial function, reduction of deoxysphingolipids	CKD mouse (podocyte senescence)	Preclinical	Hu et al. (2025)
Dietary	Low aromatic amino acid diet	Reduction of p‐cresol sulfate and indoxyl sulfate	CKD mouse	Preclinical	Barba et al. (2021)
Dietary	Low‐protein diet + ketoacids	Reduction of glomerular hyperfiltration and uremic toxins	CKD patients	Clinical (established)	Barba et al. (2021)
Supplementation	Intravenous balanced amino acids	Recruitment of renal functional reserve; provides substrates for TCA anaplerosis and ATP generation	Cardiac surgery patients (AKI prevention)	Phase III clinical	Landoni et al. (2024)
Pharmacological	BDK inhibitor (BT2)	Enhancement of BCAA catabolism via BCKDK inhibition	CKM rat models (ZSF1, SDT fatty)	Preclinical	Bollinger et al. (2025)
Pharmacological	ASNS antisense oligonucleotides	Inhibition of glutamine‐dependent anaplerosis	ADPKD mouse	Preclinical	Clerici et al. (2024)
Pharmacological	PKM2 activator (TEPP46)	Restoration of glucose oxidative phosphorylation, inhibition of DDIT3‐mediated apoptosis	DKD mouse	Preclinical	Zhao, Sun, et al. (2025)
Pharmacological	SGLT2 inhibitors (empagliflozin, dapagliflozin)	Reduction of gut‐derived uremic toxins; SAM‐dependent epigenetic repression of inflammatory genes	CKD and DKD patients	Phase III clinical	Maekawa et al. (2025)
Pharmacological	AhR antagonists (e.g., CH223191)	Blockade of IS‐induced AhR activation and PGC1α degradation	CKD mouse	Preclinical	Xie et al. (2024)
Pharmacological	Mitochondria‐targeting bioactive compounds (resveratrol, coenzyme Q10, polyphenols)	Preserve mitochondrial integrity and function; modulate SIRT1‐PGC1α signaling; reduce oxidative stress	Preclinical CKD models	Preclinical	Cecchini et al. (2026)
Microbiome	Probiotics (Bifidobacterium longum)	Reduction of serum indoxyl sulfate via indole salvage to ILA	Dialysis patients	Small clinical	Yong et al. (2024)
Microbiome	Dietary fiber supplementation	Shift of tryptophan metabolism from indole to ILA and IPA via bacterial cross‐feeding	CKD / Hemodialysis patients	Clinical + Meta‐analysis	Sinha et al. (2024)
Postbiotic	Indole‐3‐propionic acid (IPA) supplementation	Activation of SIRT1‐PGC1α signaling, enhancement of mitochondrial biosynthesis and antioxidant defense	DKD mouse	Preclinical	Zeng et al. (2025)

*Note*: “Preclinical” refers to studies in animal models; “clinical” refers to studies in human subjects; “Phase III clinical” indicates large‐scale randomized controlled trials.

Abbreviations: ADPKD, autosomal dominant polycystic kidney disease; AhR, aryl hydrocarbon receptor; AKI, acute kidney injury; ASNS, asparagine synthetase; ASO, antisense oligonucleotide; BCAA, branched‐chain amino acid; BCKDK, branched‐chain ketoacid dehydrogenase kinase; BDK, branched‐chain ketoacid dehydrogenase kinase; CKD, chronic kidney disease; CKM, cardiorenal metabolic syndrome; DKD, diabetic kidney disease; ILA, indole‐3‐lactic acid; IPA, indole‐3‐propionic acid; IS, indoxyl sulfate; PKM2, pyruvate kinase M2; SAM, S‐adenosylmethionine; SGLT2, sodium‐glucose cotransporter 2; UUO, unilateral ureteral obstruction.

## 5 UNRESOLVED QUESTIONS AND FUTURE DIRECTIONS

Several key questions remain unanswered. First, cell‐type specificity of metabolic reprogramming needs to be addressed using single‐cell and spatial metabolomics approaches (Hu et al., 2023). Second, sex differences in amino acid metabolism remain understudied; most preclinical studies still use male animals exclusively (Harris & Weiner, 2021; Hernandez et al., 2024; Mauvais‐Jarvis et al., 2017). Third, the translation of metabolic interventions to humans requires rigorous validation of off‐target effects and safety profiles (Chowdhury et al., 2023; Kim et al., 2026). Fourth, the interplay between amino acid metabolism and other nutrients needs to be integrated into therapeutic strategies (Knol et al., 2024). Fifth, biomarker development for patient stratification is critical; urinary BCAAs, serum kynurenine/tryptophan ratio, and D‐serine are promising (Hesaka et al., 2019; Lee et al., 2020; Liu et al., 2023, 2026). Sixth, the safety and efficacy of combination therapies targeting multiple amino acid pathways require further investigation.

## 6 CONCLUSION

A primary role of the kidney is the elimination of metabolic waste products while preserving whole‐body homeostasis. Yet, as discussed throughout this Review, amino acid metabolism is deeply integrated into many pathophysiological events within the kidney, encompassing tissue maintenance, regenerative processes, and disease evolution. Investigations over the past ten years have underscored the indispensable contributions of the kidney to protein and amino acid turnover, ranging from serine and arginine production to amino acid interconversion and the detoxification of potentially harmful metabolites. While many mechanistic insights derive from preclinical models, they collectively point toward a causative role of amino acid dysregulation in disease evolution, a notion that is increasingly supported by translational and clinical studies.

Different kidney disorders share common features in their metabolic abnormalities, making a systems‐level perspective essential for deciphering how these pathways interact with one another. In ADPKD, glutamine and asparagine become essential for cystogenesis. In DKD, BCAAs drive podocyte injury through PKM2‐mediated metabolic reprogramming. Tryptophan‐derived uremic solutes play a role in advancing CKD and are linked to unfavorable clinical outcomes. Emerging therapeutic strategies include dietary restriction of specific amino acids, pharmacological modulation of metabolic enzymes, microbiome‐directed interventions, and repurposing of existing drugs such as SGLT2 inhibitors. The PROTECTION trial has provided definitive clinical evidence that intravenous amino acids reduce AKI in high‐risk surgical patients (Landoni et al., 2024), opening the door for broader evaluation of amino acid‐based therapies.

Key unanswered questions, such as cell‐type specificity, sex differences, translational barriers, nutrient interactions, biomarker development, and combination therapies, should guide future research. Addressing these challenges will require interdisciplinary approaches integrating analytical chemistry, microbiomics, epigenetics, and systems biology. Ultimately, restoring amino acid homeostasis may become a cornerstone of precision medicine for patients with kidney disease.

## AUTHOR CONTRIBUTIONS

**Shuo Liu:** Conceptualization; data curation; investigation; visualization. **Caifeng Shi:** Conceptualization; formal analysis; funding acquisition; investigation. **Yang Zhou:** Conceptualization; funding acquisition; project administration; supervision. **Chunsun Dai:** Conceptualization; project administration; supervision.

## FUNDING INFORMATION

This work was supported by the Medical Research Project of Jiangsu Commission of Health (Grant No. M2025040, MQ2025008).

## CONFLICT OF INTEREST STATEMENT

The authors declare no conflicts of interest.

## ETHICS STATEMENT

No ethical approval was required for this review article, as it does not present original research involving human participants or animals.
